# Nanostructured Molybdenum Oxides from Aluminium-Based Intermetallic Compound: Synthesis and Application in Hydrogen Evolution Reaction

**DOI:** 10.3390/nano11051313

**Published:** 2021-05-17

**Authors:** Deepti Raj, Federico Scaglione, Gianluca Fiore, Federica Celegato, Paola Rizzi

**Affiliations:** 1 Dipartimento di Chimica and Centro Interdipartimentale NIS (Nanostructured Interfaces and Surfaces), Università di Torino, V. Giuria 7, 10125 Turin, Italy; deepti.raj@unito.it (D.R.); gianluca.fiore@unito.it (G.F.); paola.rizzi@unito.it (P.R.); 2 Istituto Nazionale di Ricerca Metrologica (INRIM), Str. Delle Cacce 91, 10135 Turin, Italy; f.celegato@inrim.it

**Keywords:** nanostructured materials, molybdenum oxides, free corrosion, intermetallic compound, hydrogen evolution reaction

## Abstract

Characterized by a large surface area to volume ratio, nanostructured metal oxides possess unique chemical and physical properties with applications in electronics, catalysis, sensors, etc. In this study, Mo_3_Al_8_, an intermetallic compound, has been used as a precursor to obtain nanostructured molybdenum oxides. It was prepared into ribbons by arc-melting and melt-spinning techniques. Single and double-step free corrosion of the as-quenched material have been studied in 1 M KOH, 1 M HF and 1.25 M FeCl_3_ at room temperature. In both cases, nanostructured molybdenum oxides were obtained on a surface layer a few microns thick. Two of the as-prepared samples were tested for their electrocatalytic capability for hydrogen evolution reaction (HER) in 0.5 M H_2_SO_4_ giving low onset potential (−50 mV, −45 mV), small Tafel slopes (92 mV dec^−1^, 9 mV dec^−1^) and high exchange current densities (0.08 mA cm^−2^, 0.35 mA cm^−2^ respectively). The proposed nanostructured molybdenum oxides are cost-effective and sustainable due to the cheap and abundant starting material used and the simple synthetic route, paving the way for their possible application as HER electrocatalysts.

## 1. Introduction

Molybdenum is an attractive metal which is in widespread industrial usage owing to its excellent properties—high thermal and electrical conductivity, high melting point, low thermal expansion, low vapour pressure and high temperature and wear resistance [1,2,3]. As an alloying element it enhances the corrosion resistance and mechanical properties of steels [1,3]. Molybdenum compounds have an array of applications as well—gas sensors [4,5], heterogeneous catalysis [6,7], electrocatalysis [4,8], capacitors [4,9], electrochromism [10,11], photochromism [4,12], and lithium-ion batteries [13,14]. Chalcogenide derivatives of Mo have been evidently favorable as building blocks in nanomaterial design in photocatalysis and hydrogen evolution reaction (HER) [15,16]. Due to the variable oxidation states of molybdenum, tuning of the crystal structure, morphology and oxygen vacancy is facilitated, which makes its oxide compounds suitable for electrochemical activities. Synthesis and analytical detection of numerous vital molecules has been successfully achieved using molybdenum oxide-based heterogeneous catalysis [7,17]. As gas sensors, molybdenum oxides have been well applied to NO, NO_2_, CO, H_2_, NH_3_, and other gases [1,5]. MoO_3_ is a versatile compound with noteworthy applications in electronics, catalysis, sensors, energy-storage units, biosystems, superconductors, lubricants, thermoelectric and electrochromic systems, etc. [18,19].

As a clean, efficient and sustainable alternative to fossil fuels, hydrogen gas (H_2_) has emerged to be of great importance for the future of energy generation and storage [20,21]. However, production of H_2_ from the electrocatalytic hydrogen evolution reaction (HER) still remains a challenge and has been intensively explored using a range of materials [22,23]. So far, noble metals, such as platinum, have shown the best performance as HER catalysts [20,24]. Nevertheless, the scarcity, substandard stability and high cost of these metals have been hugely limiting their usage [25,26]. Therefore, developing inexpensive earth-abundant electrocatalysts for hydrogen evolution, with increased activity and stability, is very significant in the field of clean energy [20]. Due to the extensive variations and tunable properties, molybdenum-based materials have garnered rising interest in the electrocatalytic HER [27,28]. Molybdenum oxides [20,29] and other compounds such as Mo_2_C [30], MoSe_2_ [31], MoN [32] and MoS_2_ [33,34] have been the focus of active investigation as HER electrocatalysts with MoS_2_ being the most broadly studied which exhibited favourable catalytic activity.

Nanostructured metals and metal oxides possess enhanced surface area and augmented chemical and physical properties [35,36]. The main and easy route to obtain a nanostructured material starting from an alloy is the chemical or electrochemical etching conducted by means of aqueous media. In accordance with the alloy’s initial composition and the applied condition, i.e., type, concentration, pH and temperature of the electrolyte, time of treatment, corrosion can be classified as general corrosion when the alloy is dissolved completely in the etching solution or selective when just one component of the alloy is maintained after the etching. The selective etching process can cause the dissolution of the less noble element and the oxidation of the other and, in some cases the formation of a nanostructured oxide of the remaining element; this is due to the etching condition in conjunction with the natural tendency of elements, such as molybdenum, chromium and aluminium [3], to easily passivate and react with the oxygen present in the environment. In particular, Al is dissolved during the selective corrosion of Mo-Al binary systems while Mo oxidizes and formation of a continuous passive layer occurs over the surface. This passive oxide film is superiorly stable in acidic pH and loses its stability as the pH of the solution rises due to the formation of soluble surface species [1,37,38]. In other words, due to the formation of a highly protective oxide film, acidic electrolytes are comparatively less corrosive to molybdenum while alkaline media allows for continuous dissolution of molybdenum oxides [1,39].

Herein we report the synthesis of nanostructured molybdenum oxides from a binary intermetallic compound, Mo_3_Al_8_, with the composition 28 at. % Mo and 72 at. % Al. Selective single-step and double-step free corrosion have been performed in various electrolytes with different concentrations, namely 1 M KOH, 1 M HF and 1.25 M FeCl_3_ at room temperature playing with the time of the treatment. Taking advantage of their increased surface area and typically high number of active sites, the molybdenum oxide samples obtained have been tested as non-precious electrocatalysts for the HER. The materials reported in this paper have been obtained using comparatively cheap and abundant precursors and a simple, fast and sustainable synthetic route that do not involve the use of critical raw materials. They are characterised by a good overall HER activity, paving the way for possible application of these materials as HER electrocatalysts as cost-effective alternative to Pt and Pt-based electrocatalysts. Being free-standing and easy-to-handle materials, they can potentially be economical and sustainable candidates for large-scale industrial production.

## 2. Materials and Methods

Mo_3_Al_8_ master alloy of nominal composition 28 at. % Mo and 72 at. % Al was prepared by arc-melting bars of pure elements (99.999% Mo and 99.999% Al) after evacuating and purging the furnace several times in Ti-gettered Ar atmosphere. The ingot was melt-spun from a boron nitride crucible (Edmund Bühler GmbH, Bodelshausen, Germany) at a linear speed of 25 m s^−1^ onto a hardened Cu wheel in a closed chamber kept under a protective Ar atmosphere.

Different trials of chemical and electrochemical corrosion were performed using electrolytes, namely 1 M KOH, 1 M HF. 1.25 M FeCl_3_, 1 M H_2_SO_4_, 1 M Na_2_S and piranha solution (3 parts H_2_SO_4_: 1 part H_2_O_2_) for different durations at room temperature and 70 °C. The samples reported in this paper were obtained from single and double-step free corrosion of the as-quenched alloy ribbon performed at room temperature for durations ranging from 1 h to 24 h in 1 M KOH (20 mL solution of 1.122 g KOH), 1 M HF (20 mL solution of 0.725 mL HF from a 27.59 M stock solution) and 1.25 M FeCl_3_ (20 mL solution of 3.244 g FeCl_3_). All electrolytes were prepared from chemical grade reagents and deionized water. This synthesis was repeated several times in order to check the reproducibility and the same results were always obtained.

Samples were analyzed before and after treatments using a Panalytical X-pert X-ray Diffractometer in Bragg–Brentano geometry (Panalytical, Almelo, The Netherlands) with monochromatic Cu Kα radiation, scanning electron microscopy (Inspect SEM, FEI) (FEI, Hillsboro, OR, USA), field-emission scanning electron microscopy (FIB-FESEM/EBSD/EDS/TOF-SIMS Tescan S9000G microscope) (TESCAN, Brno, Czech Republic) and energy-dispersive X-ray spectroscopy (Oxford Ultim-Max 100 connected with the FESEM) (Oxford Instruments, Abingdon, UK).

The electrocatalytic activity of the obtained samples towards HER was evaluated in 0.5 M H_2_SO_4_ aqueous solution at room temperature, using a three-electrode cell (saturated Ag/AgCl double-bridge reference electrode, Pt-grid counter and the sample as the working electrode). Linear sweep voltammetry (LSV) was performed at 2 mV s^−1^ for comparison with the data from the literature [40]. A sheet of pure Pt (2 cm^2^) was polished on the surface as per conventional metallography and used for comparison with the samples in the same experimental conditions. All potentials were reported to the reversible hydrogen electrode (RHE) adding a value of (0.199 + 0.059 pH) V. The electrochemically active surface area of the samples (ECSA) was estimated using the double layer capacitance method [20,41,42]. All measurements were performed in 0.5 M H_2_SO_4_ solution using a three-electrode cell similar to that utilized for the HER tests. Cyclic Voltammetry (CV) CV curves were obtained in the potential range of 100 mV around the open circuit potential (OCP), assuming that only non-Faradaic processes take place at the electrode-solution interface in this potential range, at scan rates of 10, 20, 30, 40, 50, 70, 90 and 100 mV/s. Then, at the middle of this potential range, the current obtained in the middle of each cycle (i.e., average of the cathodic current and the anodic current) was plotted against the scan rates mentioned above. The plots obtained are provided in the Supplementary Material (Figure S3). The current density was then normalized using the ECSA obtained for each sample.

## 3. Results and Discussion

**Figure 1:**
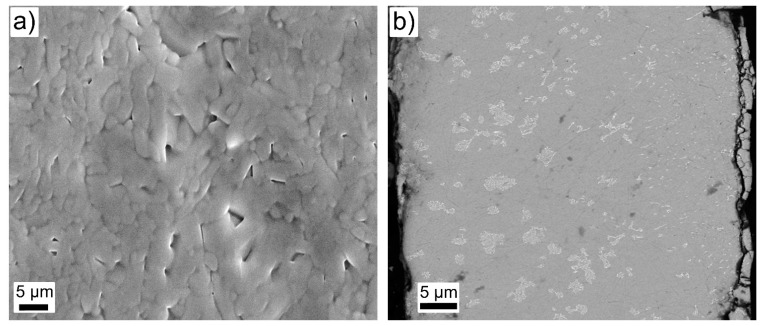
(**a**) Scanning electron microscopy-Secondary electron (SEM-SE) image of surface view of as-prepared ribbon and (**b**) SEM-back-scattered electron (BSE) image of mirror-polished cross-section of as-prepared ribbon.

Figure 1a shows the SEM secondary electron image of the as-quenched ribbon surface that exhibits a surface morphology with narrow sheet or plate-like structures woven together and overlapped closely. This morphology can be related to the shape of the crystalline grains as they grow on the surface during rapid solidification. Therefore, the presence of grain boundaries separating neighboring crystals is responsible for the roughness observed in Figure 1a. From this SEM image (Figure 1a) it is possible to estimate the crystals dimension that is on average below 10 µm. This particular morphology is of interest for the next steps of synthesis of the nanostructured molybdenum oxides being roughness and grain boundaries easy places for chemical etching.

Two phases were found to be present in the mirror-polished cross-section of the as-prepared ribbon, i.e., Mo_3_Al_8_ and AlMo_3_, as observed in the back-scattered SEM images in Figure 1b, where the darker phase can be attributed to Mo_3_Al_8_ due to its lower average atomic number, while the lighter phase is related to AlMo_3_. The amount of AlMo_3_ in Figure 1b is limited, meaning the composition of the master alloy ingot was just slightly above than what is required for obtaining only the linear compound Mo_3_Al_8_. The microstructure present in Figure 1b can be clearly attributed to the formation of Mo_3_Al_8_ with a peritectic reaction at high temperature, followed by a eutectoid reaction at 1838 K being present areas in which alternated lamella of Mo_3_Al_8_ and AlMo_3_ are visible.

**Figure 2:**
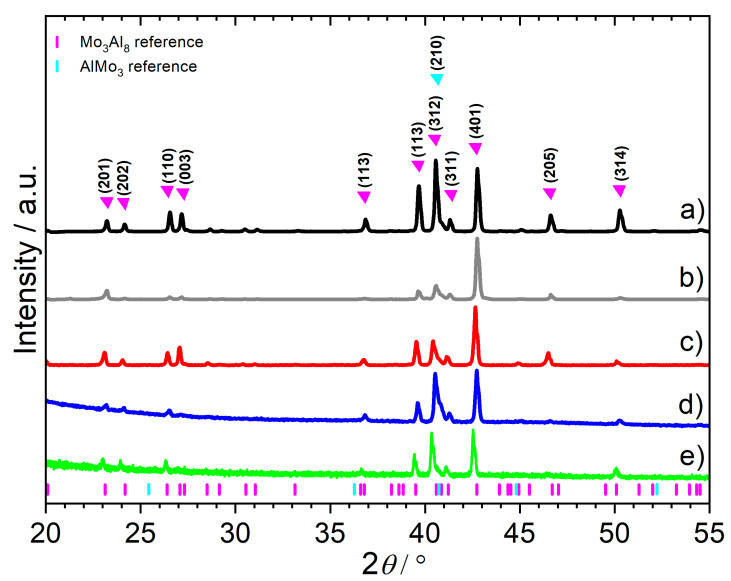
X-ray diffraction (XRD) pattern of (**a**) the wheel-side of the as-quenched ribbon; (**b**) the air-side of the as-quenched ribbon; (**c**) the air side of the sample obtained by single-step free corrosion in 1 M KOH at room temperature for 6 h (denoted as SS_KOH); (**d**) wheel side of the sample obtained by single-step free corrosion in 1 M HF at room temperature for 24 h (denoted as SS_HF) and; (**e**) wheel side of the sample obtained by double-step free corrosion first in 1.25 M FeCl_3_ for 1 h and then in 1 M HF for 6 h both at room temperature (denoted as DS_1h).

The XRD data in Figure 2a show the typical patterns associated with Mo_3_Al_8_ intermetallic phase obtained from the wheel-side of the as-quenched ribbon. Phase determinations were made using Standard ICDD (International Centre for Diffraction Data) card no. 03-065-6867 for Mo_3_Al_8_ and 03-065-4685 for AlMo_3_. A textural effect at (−312) and (401) reflections are observed in the pattern taken from the air-side (Figure 2b): solidification front starts in the region of the ribbon which is in direct contact with the wheel of the melt- spinning apparatus and then proceeds in the direction along which the heat is subtracted during the rapid quenching process, causing an orientation of crystals.

**Figure 3:**
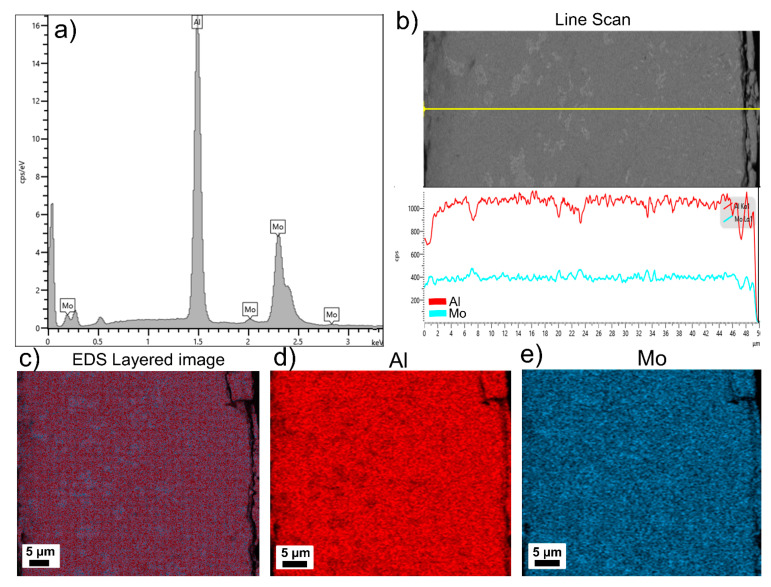
Energy-dispersive X-ray spectroscopy (EDS) analyses of the cross-section of the as-quenched ribbon: (**a**) EDS spectrum; (**b**) Line scan—red signal for Al and cyan signal for Mo; (**c**) Layered compositional map depicting Mo and Al content; (**d**) Map showing only Al content and; (**e**) Map showing only Mo content.

The composition of the as-spun ribbon was carefully analyzed by means of EDS, performing average spot analyses, compositional maps and line scans. The EDS spectrum (Figure 3a) presents the main Al and Mo peaks; the average composition was determined to be 32 at. % Mo and 68 at. % Al with a standard deviation of 3 at. %. The EDS compositional maps show a widespread and uniform presence of Al and Mo throughout the surface of the as-spun ribbon. Obviously, more Mo was found in the AlMo_3_ minority phase. The line scan data (Figure 3b) portrays a uniform trend in the Al and Mo intensity signals interrupted by a jump in the composition when the scanning line intercepts the AlMo_3_ phase. This observation further supports the findings from the SEM images and XRD analyses described above.

### 3.1. Free Corrosion

**Scheme 1:**
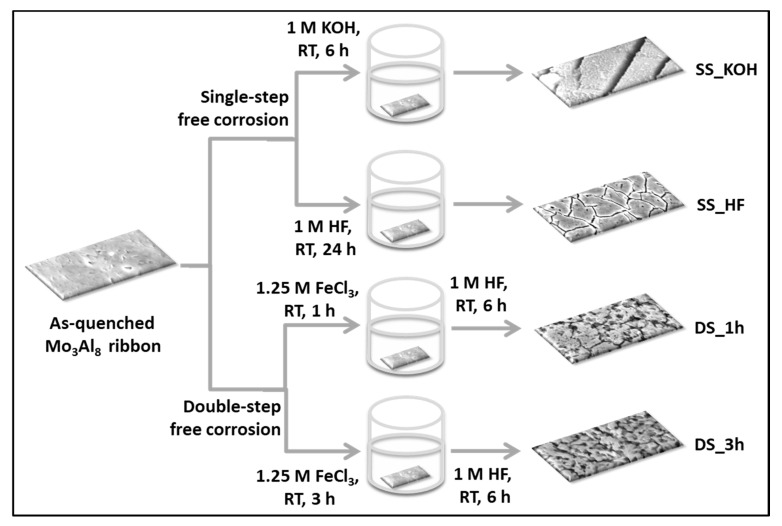
Scheme demonstrating the overall free corrosion process.

A number of trials of chemical and electrochemical corrosion were performed using an array of electrolytes for different durations at room temperature and 70 °C, as mentioned above in the Materials and Methods section. Some of the obtained samples suffered from an excess of embrittlement and the SEM images can be found in Figure S1 of the Supplementary Material. These samples were not further studied being the goal of this study to propose mechanically stable, free-standing and easy-to-handle materials. In this respect, the most promising samples, in terms of mechanical stability and desired composition, turned out to be the ones prepared by single-step and double-step free corrosion using KOH, HF and FeCl_3_ at room temperature varying the time of the treatment as demonstrated in Scheme 1. KOH and HF were selected as the etching electrolytes for selective corrosion of Al. FeCl_3_ was used in the first step of the double-step free corrosion treatment to add another level of corrosion and facilitate nanostructuration in the morphology of the ribbon.

From here onwards the samples have been denoted in the following manner—the sample prepared by single-step free corrosion in 1 M KOH at room temperature for 6 h is SS_KOH; the sample prepared by single-step free corrosion in 1 M HF at room temperature for 24 h is SS_HF; the sample prepared by double-step free corrosion first in 1.25 M FeCl_3_ for 1 h and then in 1 M HF for 6 h both at room temperature is DS_1h and; the sample prepared by double-step free corrosion first in 1.25 M FeCl_3_ for 3 h and then in 1 M HF for 6 h both at room temperature is DS_3h.

#### 3.1.1. Single-Step Free Corrosion

With the intent to remove the maximum amount of Al and induce the nanostructural roughening in the whole thick ness of the sample, free corrosion was performed in 1 M KOH for 6 h at room temperature and the obtained sample was characterized.

**Figure 4:**
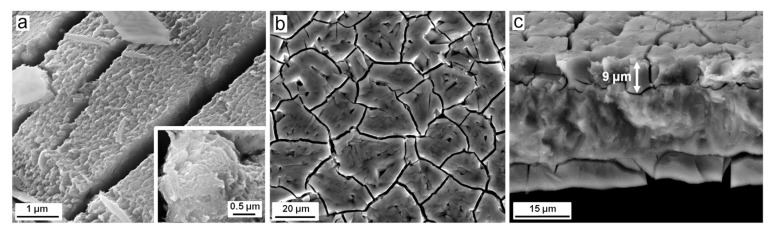
SEM images of (**a**) surface view of SS_KOH, (**b**) surface view of SS_HF and (**c**) cross-sectional view.

The XRD pattern of the air-side of SS_KOH (Figure 2c) resembles that of the air-side of the as-quenched ribbon (Figure 2b). The surface of the as-treated sample, SS_KOH (Figure 4a) is divided into crack patterns of micrometric size. Cracks could be due to stress corrosion and differences in volume between the pristine intermetallic alloy and the formed oxides. The inset of Figure 4a reports a magnified image of the sample surface where a rough morphology can be observed, spread from the top of the patterned surface and extended only up to a few microns inside the cross-section. As for the rest of the cross-section, no nanostructured morphology was observed. From the compositional analysis by EDS, it is revealed that the ratio Al/Mo on the surface and in the cross-section of the as-treated sample is similar to the ratio Al/Mo of the as-quenched ribbon. As a result, no selective corrosion was achieved but a general corrosion with the formation of a scale composed by mixed Al and Mo oxides. By contrast, in XRD, just the pristine intermetallic alloy was observed. This could be explained by the low scattering factor of oxides with respect to metals and to the low symmetry of the Mo_3_Al_8_ phase. On one hand the oxides have a low scattering intensity with respect to metals, while on the other, weak peaks related to the oxide phases can be superimposed by the high number of peaks related to the Mo_3_Al_8_ phase.

The effect of a different electrolyte was studied by using 1 M HF. High temperature treatment was avoided as it would be too harsh for the sample causing brittleness and worsening its stability. Accordingly, free corrosion was performed at room temperature in 1 M HF for 6 h but no appreciable formation of Mo oxides was obtained. When the treatment time was increased to 24 h, the surface of the resultant sample (named SS_HF) was observed to be cracked, as already seen in the previous case, but inside the patterned region a compact layer of Mo oxides is present (Figure 4b). This cracked pattern is not extended in the whole cross-section of the ribbon but only up to about 9 microns approximately (Figure 4c).

**Figure 5:**
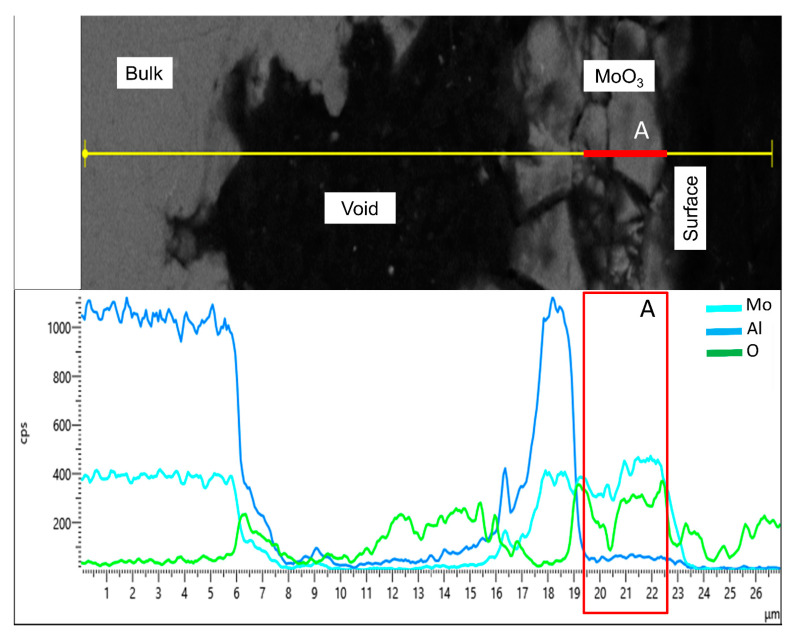
EDS line scan of SS_HF: blue signal for Al, cyan for Mo and green for O.

**Figure 6:**
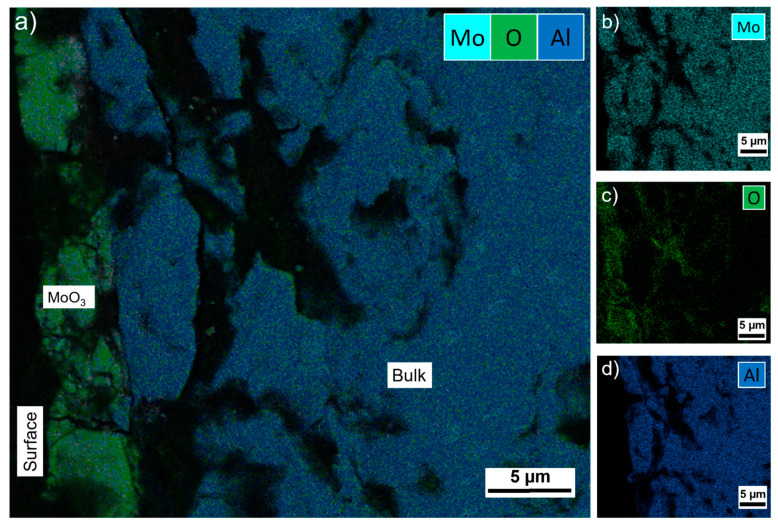
EDS analysis of SS_HF: (**a**) layered compositional map depicting Mo, O and Al content; (**b**) Mo elemental map; (**c**) O elemental map; and (**d**) Al elemental map.

The XRD pattern shown in Figure 2d presents a likeness to that of the wheel side of the as-quenched ribbon (Figure 2a) due to similar reason explained for the previous sample. In the EDS line scan in Figure 5 it can be seen that starting from the left part of the cross-sectional image the counts per second (cps) signals for Mo (in cyan) and Al (in blue) maintain their intensity as the scan continues along the thickness of the sample represented by the yellow line on the cross-sectional image. As soon as a void is encountered due to the inhomogeneity of the sample, there is a fall in both the signals. After crossing the void area, both the signals jump to their usual intensities. Towards the outer edge of the cross-section, highlighted by the red line segment A, oxygen signal in green comes into the picture (within the red box A). Mo signal is slightly enhanced while Al signal is reduced drastically. This suggests the formation of the Mo oxide. In Figure 6a the compositional analysis of the cross-section of SS_HF done by EDS has been shown in the form of mappings. It can be seen that the selective corrosion has only taken place on the outer edge of the cross-section (highlighted by a mix of green and cyan colours) which represents the surface of the sample. A prominent attendance of oxygen is displayed in the corroded region along with the abundance of Mo. This implies that this corroded edge is composed of Mo oxide. Also, there are no notable traces of Al in this corroded layer. And, the bulk of the cross-section lacks any signs of corrosion as the Al and Mo compositions are not depleted and oxygen is absent. The EDS analysis shows the presence of MoO_3_ on the edges. Figure 6b–d display the elemental maps of Mo, O and Al further clarifying the scenario in which Mo and O are prominently present on the outer edge while Al has disappeared.

**Figure 7:**
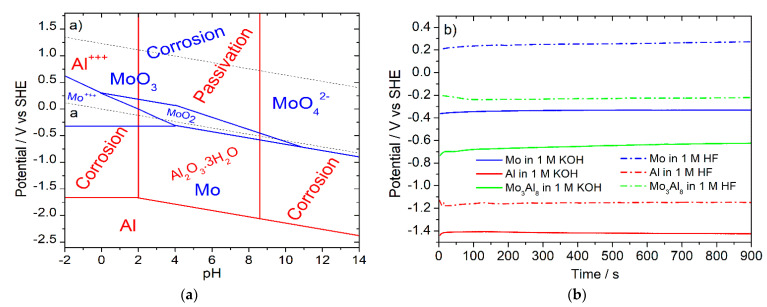
(**a**) Superimposed Pourbaix diagrams of pure Al and Mo showing the regions of stability, passivation and corrosion of each element. (**b**) Evolution of the open-circuit potential of pure Al and Mo, as references, and the as-quenched Mo_3_Al_8_ ribbon.

The explanation for the behaviour observed in both the above samples, i.e., SS_KOH and SS_HF, can be attributed to the specific reactivity of Al and Mo in the experimental conditions applied for the selective corrosion treatment, i.e., 1 M KOH (pH = 14) and 1 M HF (pH = 1.56) respectively. A superimposed Pourbaix diagram showing the regions of stability, passivation and corrosion of both elements has been reported in Figure 7a [1]. It depicts that Mo dissolves in aqueous solutions with neutral-to-alkaline pH due to the amplified stability of molybdenum oxyanions [3,43]. In a strongly basic condition and at a potential higher than −0.89 V vs. SHE, Mo is unstable in the metallic state and is converted to molybdate (MoO_4_^2−^) ion while for a wider range of potential Al is fully corroded. Figure 7b shows the open-circuit potentials vs SHE of the pure Al and Mo, and the as-quenched Mo_3_Al_8_ ribbon while the open-circuit potentials vs RHE for the same can be found in Figure S2 in the Supplementary Material. In 1 M KOH (Figure 7b, solid lines) the open-circuit potentials are found to be 1.42 V, −0.33 V and −0.62 V, respectively, for pure Al and Mo, and as-quenched ribbon meaning that the pure elements and the ribbon are all in the condition of corrosion. Moreover, molybdenum is only weakly resistant to hydroxides [3] and oxidizing alkalis convert it into molybdate [44]. Hence, the morphology observed under alkaline etching in case of SS_KOH, in agreement with XRD and EDS results, is due to a general corrosion rather than a selective corrosion process.

In the case of SS_HF, Figure 7b represents the OCP curves obtained for pure Al and Mo, and the as-quenched Mo_3_Al_8_ ribbon in 1 M HF in dot-dashed lines. The OCP value of −1.15 V for pure Al lies in the corroded region of the Pourbaix diagram and that of 0.28 V for pure Mo lies in the oxidized region. The Pourbaix diagram shows that in aqueous solutions with acidic pH, Mo forms passive oxides on its surface [3,43]. While Al is corroded in the strongly acidic condition (pH ≤ 2), a value of E_ocp_= −0.22 V vs. SHE measured for the as-quenched ribbon falls in the region of Mo^3+^ ions. This means that being in acidic media, at first Mo^3+^ ions are formed at relatively low electrode potentials and then as the reaction proceeds the surface is covered with a passivating layer of MoO_2_ or MoO_3_ [44]. In addition, molybdenum does not dissolve appreciably in non-oxidizing acids [44] and is relatively unaffected by the presence of halide ions, relatively resistant to most localized corrosion processes [3]. Therefore, it has good performance in hydrohalide acids, i.e., HF in this case [3]. As a consequence, selective corrosion takes place where Al is dissolved on the top surface while Mo undergoes oxidation and forms a compact passive oxide layer [37,43] on the terraces which results in the formation of nanostructured molybdenum oxides.

#### 3.1.2. Double-Step Free Corrosion

The double-step free corrosion was undertaken with the intention that applying dedicated individual steps of general and selective corrosion would result in a Mo oxide-rich product with improved nanostructural morphology and surface area. The first step of the treatment was particularly dedicated to corrode and expose the surface using a corrosive electrolyte. This would cause roughness and simultaneously remove stable Al and Mo oxides covering the sample surface that can impede the dissolution process. The second step was targeted to remove the Al content via selective corrosion with the help of a suitable electrolyte.

For the first step, FeCl_3_ was chosen which is a well-known corrosive agent and has been reported in literature as being successfully used as an etchant for Al and its alloys [45]. In addition, oxidizing conditions severely reduce molybdenum’s corrosion resistance, and aeration causes a significant boost in corrosion [3]. Therefore, FeCl_3_, being a reducing acid containing oxidizer, rapidly attacks molybdenum.

The as-quenched ribbon was treated in 1.25 M FeCl_3_, chosen from literature [45], at room temperature from 1 h to 8 h. Only the samples obtained after 1 h and 3 h of treatment were taken into account as the rest suffered from brittleness due to the prolonged treatments. However, the observed pitting corrosion was limited to the surface of the obtained samples.

**Figure 8:**
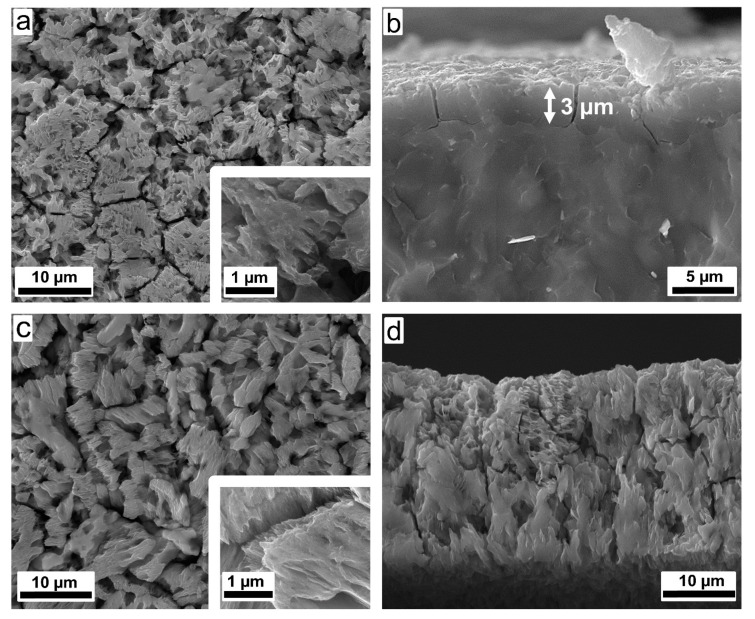
SEM images (on the left—surface view; on the right—cross-sectional view) of (**a**,**b**) DS_1h and (**c**,**d**) DS_3h.

**Figure 9:**
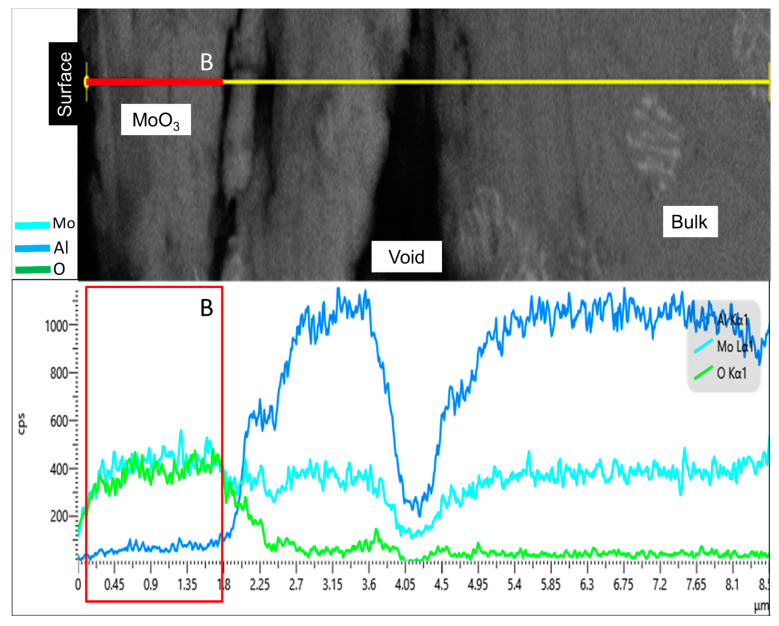
EDS line scan of DS_1h: cyan for Mo, blue signal for Al and green for O.

**Figure 10:**
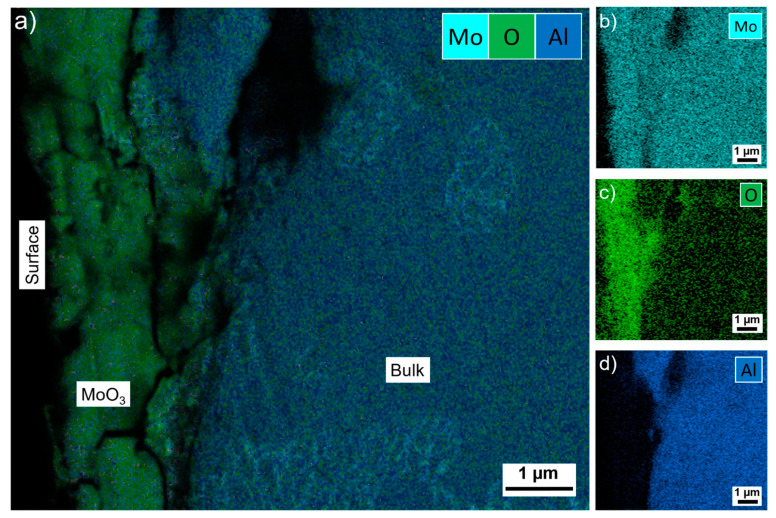
EDS analyses of DS_1h: (**a**) layered compositional map depicting Mo, Al and O content; (**b**) Mo elemental map; (**c**) O elemental map and; (**d**) Al elemental map.

Considering the effect of HF in eliminating high amounts of Al observed for sample SS_HF as previously described, 1 M HF was used for the second step and the free corrosion was performed for 6 h at room temperature for the samples treated for 1 h and 3 h, namely DS_1h and DS_3h respectively. From the SEM images of DS_1h (Figure 8a,b) it can be observed that the sample acquired an inhomogeneous porosity as a result of pitting corrosion. After 6 h of treatment in HF, the sample was affected by the electrolyte on a surface layer of 3 µm. The composition was measured by the EDS which showed the presence of MoO_3_ on the surface. Figure 9 presents the line scan analysis of the cross-section in which the trends are similar to that already seen in the case of SS_HF. The cps signals for Mo (in cyan) and O (in green) are visible in the outer region of the cross-section (highlighted by the red line segment B and red box B) with negligible Al signal indicating the presence of Mo oxide. Continuing the scan, the oxide layer is passed by and the pristine phase appears which is demonstrated by the fall in O signal and simultaneous emergence of Al signal along with a slight decrease in Mo signal. The signals drop throughout the existence of the void which rise back once the void is crossed and the pristine phase prevails. The Mo signal in cyan retains its intensity throughout the cross-section demonstrating no significant decrease in the Mo concentration in the volume of the sample, suggesting that Mo is resistant to dissolution by HF and, rather, forms a passive oxide layer. It other words, the chemical etching using 1 M HF did not reduce the concentration of Mo but Al content has been notably removed by the etching treatment in a 3 µm layer where Mo oxides have formed. Thus, HF has only acted upon Al, as intended, selectively eliminating it and facilitating formation of Mo oxides. As analysed earlier in Section 3.1.1. ‘Single-step free corrosion’, the Pourbaix diagram (Figure 7a) validates this observation. Based on the OCP values of pure Al and Mo from Figure 7b, i.e., −1.15 V and 0.28 V vs SHE respectively, pure Al lies in the corroded region of the diagram while pure Mo lies in the oxidized region. This means that treatment of the as-quenched ribbon (OCP = −0.22 V) with 1 M HF results in selective corrosion with Al being corroded while Mo undergoes oxidation forming a passivating layer of nanostructured Mo oxides. Figure 10a shows the Mo-oxide-rich outer-region of the cross-section highlighted by the mix of cyan and green signals for Mo and O respectively. Figure 10b–d display the elemental maps of Mo, O and Al that add up to the overall observation of the presence of Mo oxide on the sample surface. The bulk of the cross-section is protected from the corrosion as shown by the unchanged Al and Mo compositions, the Mo and Al signals in the line scan and the elemental maps.

In the case of DS_3h, the SEM images (Figure 8c,d) display a more homogeneous nanostructural surface with nanoplates. This can be linked to the extended duration of the first step of the corrosion process that increased the depth of etching and facilitated constant and stable treatment [45]. For the same reason a slightly nanostructural morphology can be noticed in the cross-section as well. The EDS results confirm the high content of Mo oxide on the surface by the action of HF in the second step. Observing this carefully, it can be inferred that the resultant microstructure resembles the as-quenched ribbon morphology. The corrosion must have started around the boundaries of the plate-like grains and the defects on the surface of the as-quenched ribbon (as shown in Figure 1 earlier). As a result, there is the formation of nanoplates along with grooves in the microstructure. The overlapping and compactness have been reduced compared to that in the as-quenched ribbon. The nanoplates also pose a roughened and layered texture increasing their surface area.

From the XRD data of DS_1h (Figure 2e) it is revealed that the obtained pattern largely mimics that of the as-quenched ribbon (Figure 2a). This is due to the negligible contribution of the 3 µm layer of Mo oxide to the XRD pattern as it has been overlapped by the major contribution of the Mo_3_Al_8_ intermetallic phase present in the bulk and to the low scattering factor of oxides with respect to metals as previously described for the other samples. Similar results were obtained for DS_3h.

Thus, a nanostructural molybdenum oxides rich sample with enhanced surface area was successfully obtained by using a double-step free corrosion process.

### 3.2. Hydrogen Evolution Reaction (HER)

Since, Mo oxides have been proven as active electrocatalysts for HER [20,27,29], we tested our samples, SS_HF and DS_1h, for the same.

**Figure 11:**
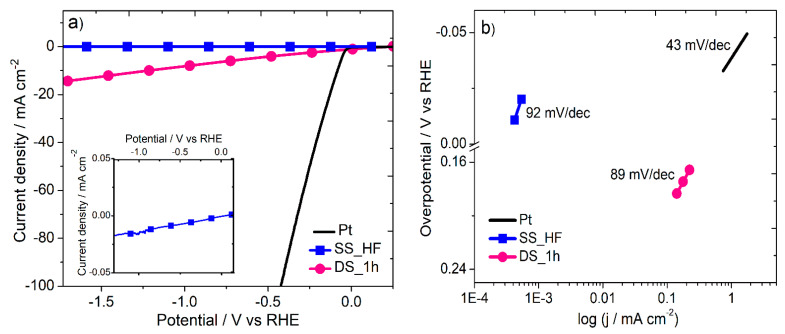

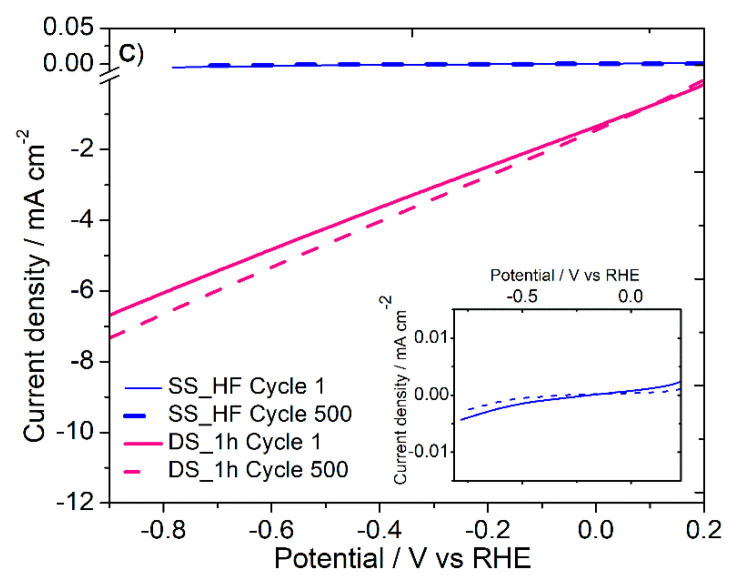
For pure Pt (solid black line), SS_HF (blue line with cubes) and DS_1h (dotted pink line) (**a**) linear sweep voltammetry (LSV) polarisation curves, inset shows the magnified view of the curve for SS_HF; (**b**) Tafel slope; and (**c**) LSV polarization curves after 500 cycles of potential scans for SS_HF and DS_1h, inset shows the magnified view of the curves for SS_HF.4.

Figure 11a reports LSV polarization curves for pure Pt, SS_HF and DS_1h. The current density involved in the whole range explored is always higher for Pt compared with that of SS_HF and DS_1h. The onset potential, which marks the onset of a large increment in current, comes out as −50 mV for SS_HF and −45 mV for DS_1h. These values top those of some already reported electrocatalysts such as −80 mV for core-shell nanocomposite based on Au nanoparticle@Zn-Fe-embedded porous carbons (Au@Zn-Fe-C) [46]; 198 mV for CoTe_2_ nanoparticles [47]; −58 mV for N-graphene/Co-embedded porous carbon derived from Metal Organic Frameworks [48]; and 82 mV for hierarchical β-Mo_2_C nanotubes [49].

DS_1h reaches the current density of −10 mA cm^−2^ at an overpotential of −1.24 V. The Tafel plots of the samples and the Pt reference were estimated in the region below the onset potential by linearly fitting data with the Tafel equation,

*η* = *blogj* + *a*

where *η* is the overpotential, j is the current density and b is the Tafel slope [50]. Tafel slope is a guide to determine the mechanism and the rate-determining step (r.d.s.) for the HER, based on the classical combination or Tafel reaction and (iii) desorption or Heyrovsky reaction [51,52]. The first step is ruled by the discharged process where a proton and a transferred electron interact, forming an adsorbed hydrogen atom on the electrode surface [53]. Then, the reaction could follow either the Tafel or the Heyrovsky reaction: in the former case, two adsorbed hydrogen atoms combine to evolve H_2_, whereas in the second case, an adsorbed hydrogen atom, a proton from the solution and another electron react to generate H_2_. The rate-determining step (r.d.s.) of the HER can be evaluated by the examination of the Tafel slope: a slope of 120 mV/dec or higher indicates that the r.d.s. is the Volmer reaction; slopes of 40 mV/dec are found when the r.d.s is the Heyrovsky reaction while when the r.d.s. is the Tafel step, slope decreases to a value of 30 mV/dec [54].

From Figure 11b, low Tafel slope values of 92 mV/dec for SS_HF and 89 mV/dec for DS_1h have been obtained signaling that Volmer is the rate-determining step [54,55]. This also suggests that with increasing applied potentials a faster surge in the HER rate will occur for both the samples. To compare with the literature, the Tafel slopes obtained in this work noticeably outdo the value of 130 mV/dec for Au@Zn–Fe–C mentioned previously [46]; 120 mV/dec for Co-doped MoS_2_ nanosheets, Co-MoS_2_-0.5 [56]; 370 mV/dec and 138 mV/dec for Pd-based nanoalloys assembled on reduced graphene oxide, rGO-Fe_48_Pd_52_ and rGO-Au_48_Pd_52_ respectively [57]; 125 mV/dec for Co-MOF, CTGU-5 [58]; 165 mV/dec reported for Au–Pd alloy nanoparticles electrodeposited on microwave assisted sol-gel-derived carbon ceramic electrodes, namely Au-MWCCE [59]; 126 mV/dec for N-graphene/co-embedded porous carbon derived from MOFs [48]; 94 mV/dec for nanostructured core–shell CoS_2_@MoS_2_/CP [60]; mV/dec for 100 mV/dec for Ni/Mo_2_C nanoparticles coated with graphene shells, NiMo_2_C@C [61]; 96 mV/dec for Co_9_S_8_/CoS_1.097_/rGO prepared from Co-MOF [62]; and 116.9 mV/dec for Ni-doped Mo_2_C coating on carbon fiber paper, Ni-Mo_2_C/CFP [63].

From the intercept of the Tafel plot high values of exchange current densities for SS_HF and DS_1h are determined as 0.08 mA cm^−2^ and 0.35 mA cm^−2^, respectively. It is known that if the exchange current density is high, the surface of the electrode is more active which means that the charge has to overcome lower energy barrier in moving from electrolyte to the catalyst surface, and vice versa [64]. Accordingly, electrochemical reaction are is fast and high current generation takes place at a given overpotential [64]. The obtained exchange current density values surpass a number of those formerly reported in literature such as, 8.32 × 10^−9^ mA cm^−2^ for the aforementioned Au-MWCCE [59]; 1.92 × 10^−3^ mA cm^−2^ for N-doped carbon coated Co–Ni alloy with reduced graphene oxide decoration (CoNi@N-C/rGO) [65]; 0.017 mA cm^−2^ for hierarchical β-Mo_2_C nanotubes [49]; 9.2 × 10^−4^ mA cm^−2^ for cocoon-like molybdenum sulfide nanostructures (MoS_2_-Mo-1h) [66]; 5.9 × 10^−5^ mA cm^−2^ for CoTe_2_ nanoparticles [47]; and 0.017 mA cm^−2^ reported for Pd-modified carbon fibre electrode [67,68]. The value of 0.35 mA cm^−2^ for DS_1h exceeds that of 0.13 mA cm^−2^ for aforementioned rGO-Au_48_Pd_52_ [57] and 0.126 mA cm^−2^ for nanostructured porous gold film [69]. Figure 11c illustrates the LSV polarization curves of SS_HF and DS_1h after 500 cycles of potential scans showing their efficient durability. Moreover, chronoamperometric measurements were also performed in 0.5 M H_2_SO_4_ applying a potential of −0.48 V vs. RHE that remains constant during the measurement (Figure S4 in the Supplementary Material). The current is found to be generally stable for a period of more than 15 h for both the samples, once again confirming their remarkable stability for HER. The XRD patterns of the samples after the chronoamperometric stability test have been provided in Figure S5 in the Supplementary Material, showing no significant differences compared to the XRD patterns obtained before the test as already provided in Figure 2. SEM images were also obtained for the samples after the chronoamperometric stability test (Figure S6 in the Supplementary Material) where no changes in the morphology can be noticed. Moreover, EDS analysis was performed and no variations in the composition was detected with respect to the as prepared sample.

Active sites for molybdenum oxides are reported in literature to be related to variations in the oxidation states of the material. The possible anion vacancies, which are viewed as the electrochemical active sites, stimulate the HER [70,71], favour the electrochemical kinetics and can also significantly increase the electrical conductivity of the electrode [72]. A similar behaviour can be inferred for SS_HF and DS_1h samples.

Based on the above results it is clear that DS_1h shows smaller onset potential and Tafel slope than SS_HF. It also gives higher exchange current density than SS_HF. Moreover, only DS_1h is capable of reaching the current density of −10 mA cm^−2^. These findings can be credited to its nanostructured morphology with larger surface area compared to that of SS_HF which increases the number of active sites enhancing the overall HER activity.

## 4. Conclusions

Nanostructured molybdenum oxides have been obtained using an intermetallic Mo_3_Al_8_ precursor formed into ribbons by arc melting and melt-spinning techniques. The precursor was subjected to single and double-step free corrosion in 1 M KOH, 1 M HF and 1.25 M FeCl_3_ at room temperature for varying durations. In the case of single-step free corrosion using KOH, the morphology, characterized by a rough surface with pores, was ascribed to general corrosion as confirmed by the EDS analysis which gave nominal composition on the sample surface and in the bulk. On treating the sample with HF, about 9 µm thick layer of MoO_3_ was obtained. However, the layer was compact and the nanostructural morphology could not be achieved. Overall, HF was found to be much more active in removing the Al content from the sample surface and cross-section as compared to KOH in case of single-step free corrosion. When it came to double-step free corrosion, one of the samples, after treatment with FeCl_3_ for 1 h and HF for 6 h, subsequently exhibited nanostructural morphology on the surface—3 µm thick layer rich in MoO_3_. None of the treatments could fully generate nanostructuration in the cross-section of the samples. However, in terms of both morphology and composition of the as-treated sample, double-step free corrosion proved to be better than the single-step. Two samples, i.e., SS_HF and DS_1h were selected to be tested as electrocatalysts for the HER in 0.5 M H_2_SO_4_. The measured values for SS_HF and DS_1h respectively are low onset potential of −50 mV and −45 mV; small Tafel slopes 92 mV dec^−1^ and 89 mV dec^−1^ indicating Volmer as the rate-determining step; and high exchange current density of 0.08 mA cm^−2^ and 0.35 mA cm^−2^. DS_1h is able to reach a current density of −10 mA cm^−2^ at an overpotential of −1.24 V. Both the samples show stability up to 15 h with no significant changes in their properties. As a whole, these are good findings considering the fact that the samples are obtainable via a fast, simple, low-cost and sustainable overall process including the starting material. The samples can be further developed to enhance their electrocatalytic activity in HER.

## Data Availability

Data will be made available on request.

## Supplementary Materials

The following are available online at https://www.mdpi.com/article/10.3390/nano11051313/s1, Figure S1: SEM images of samples obtained by different chemical and electrochemical corrosion trials; Figure S2: Evolution of the open-circuit potential vs RHE of pure Al, pure Mo and the as-quenched ribbon; Figure S3: Details of the ECSA analysis of SS_HF and DS_1h using the double layer capacitance method; Figure S4: HER stability tests for SS_HF and DS_1h by chronoamperometric method; Figure S5: XRD patterns of SS_HF and DS_1h after the chronoamperometric stability test; and Figure S6: SEM images of SS_HF and DS_1h after the chronoamperometric stability test in 0.5 M H_2_SO_4_ at −0.48 V vs RHE for 15 h.
