# Supplementary material for: Nanostructured Molybdenum Oxides from Aluminium-Based Intermetallic Compound: Synthesis and Application in Hydrogen Evolution Reaction
Source: Nanomaterials (Basel). 2021 May 17;11(5):1313. doi: 10.3390/nano11051313 (PMC8156916; doi:10.3390/nano11051313)
Supplement: Supplementary file 1 [file nanomaterials-11-01313-s001.zip › nanomaterials-1224470-supplementary.pdf]

Supplementary Information:

Nanostructured Molybdenum Oxides from Aluminium-Based Intermetallic Compound: Synthesis and Application in Hydrogen Evolution Reaction

Deepti Raj [1], Federico Scaglione [1,*], Gianluca Fiore [1], Federica Celegato [2] and Paola Rizzi [1]

[1] Dipartimento di Chimica and Centro Interdipartimentale NIS (Nanostructured Interfaces and Surfaces), Università di Torino, V. Giuria 7, 10125 Turin, Italy; deepti.raj@unito.it (D.R.); federico.scaglione@unito.it (F.S.); gianluca.fiore@unito.it (G.F.); paola.rizzi@unito.it (P.R.)

[2] Istituto Nazionale di Ricerca Metrologica (INRIM), Str. Delle Cacce 91, 10135 Turin, Italy; f.celegato@inrim.it

* Correspondence: federico.scaglione@unito.it

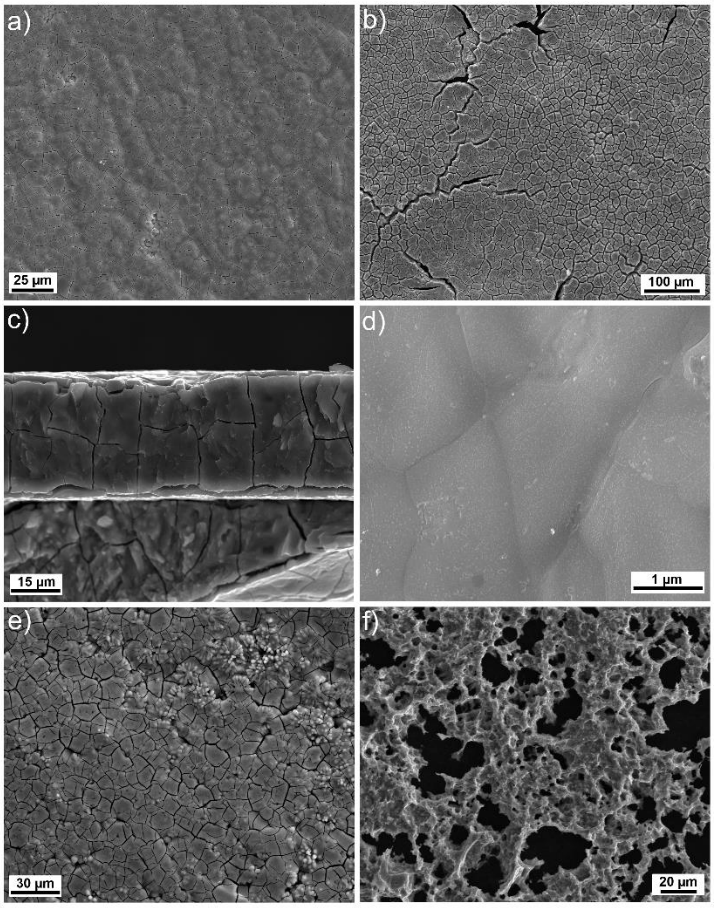

Figure S1. SEM images of sample obtained by **a**) free corrosion in 1 M KOH for 6 h at 70 °C; **b**) electrochemical corrosion at 0.8 V in 1 M KOH for 2 h at room temperature; **c**) electrochemical corrosion at -0.4 V in 1 M HF for 1 h at 70 °C; **d**) electrochemical corrosion at 0.15 V in 1 M H_2SO_4 for 1 h at 70 °C; **e**) free corrosion in 1 M Na_2S for 6 h at 70 °C; and **f**) free corrosion in piranha solution (3:1) for 18 h at room temperature.

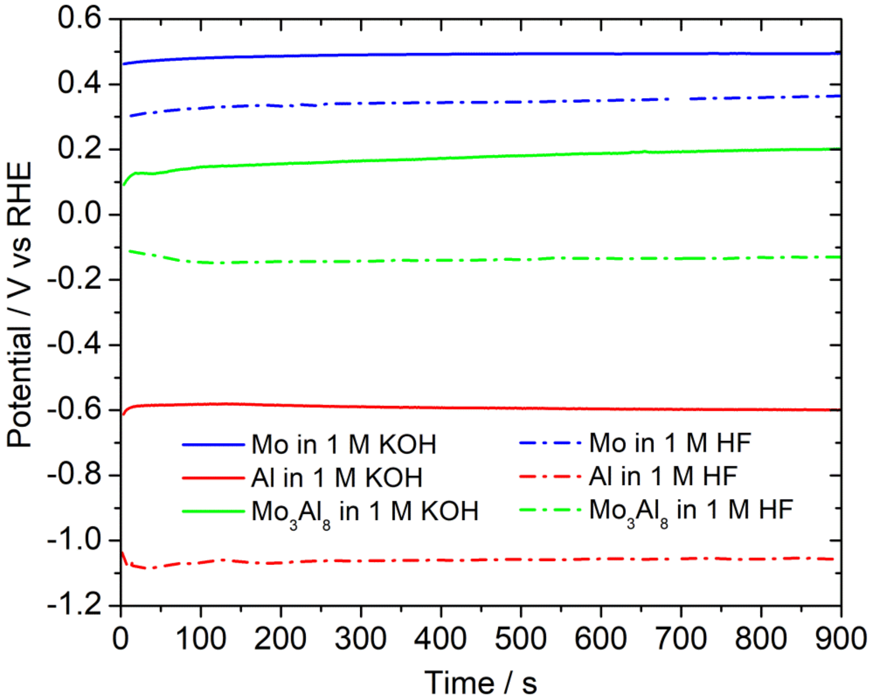

Figure S2. Evolution of the open-circuit potential (vs RHE) of pure Al and Mo, as references, and the as-quenched Mo_3Al_8 ribbon vs RHE for 15 h

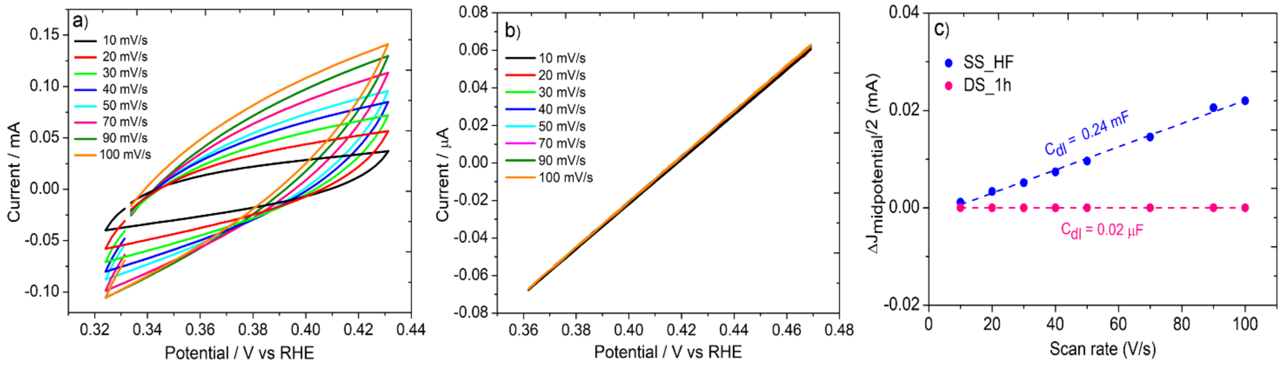

Figure S3. ECSA analysis of SS_HF and DS_1h: CV curves for **a)** SS_HF, **b)** DS_1h and **c)** corresponding dependence of $\Delta J_{midpotential}/2$ and scan rate for SS_HF and DS_1h

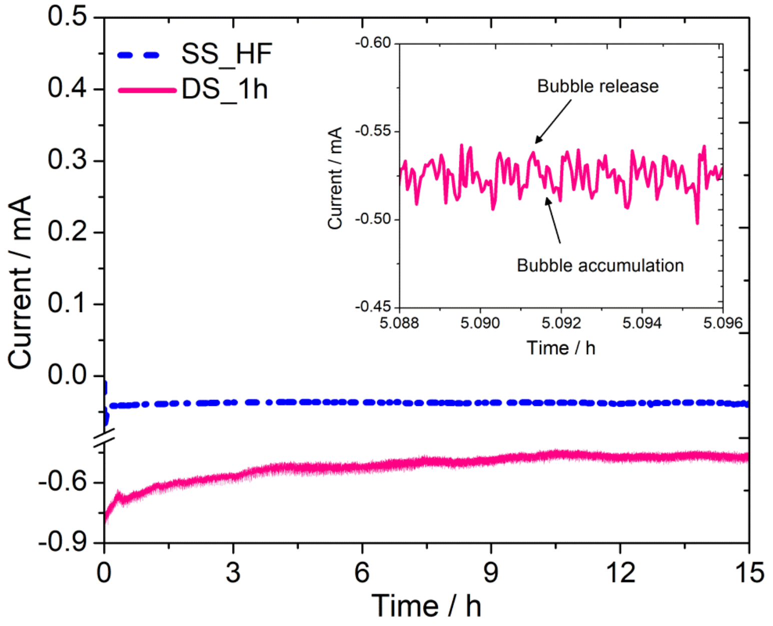

Figure S4. Chronoamperometric measurements for SS_HF and DS_1h in 0.5 M H_2SO_4 at −0.48 V vs RHE

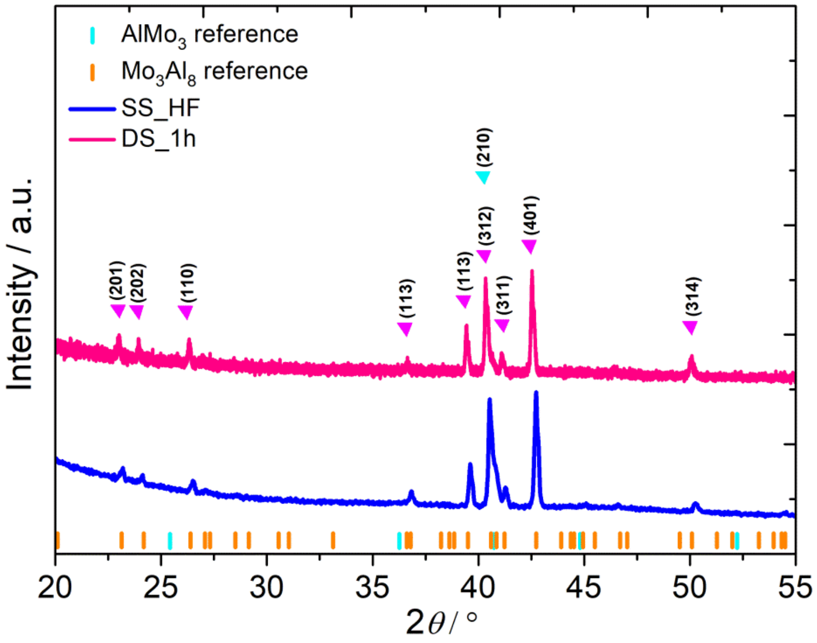

Figure S5. XRD patterns of SS_HF and DS_1h after the chronoamperometric stability test in 0.5 M H_2SO_4 at −0.48 V vs RHE for 15 h

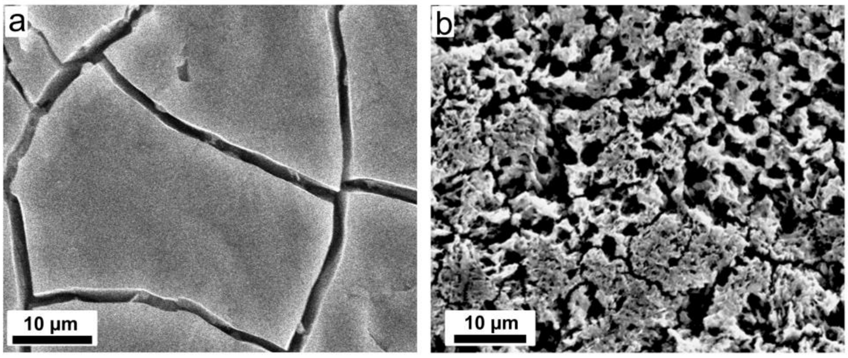

Figure S6. SEM images of SS_HF and DS_1h after the chronoamperometric stability test in 0.5 M H_2SO_4 at −0.48 V vs RHE for 15 h
